# MiR-125b-2 knockout increases high-fat diet-induced fat accumulation and insulin resistance

**DOI:** 10.1038/s41598-020-77714-7

**Published:** 2020-12-15

**Authors:** Li-Min Wei, Rui-Ping Sun, Tao Dong, Jie Liu, Ting Chen, Bin Zeng, Jia-Han Wu, Jun-yi Luo, Jia-Jie Sun, Qian-Yun Xi, Yong-Liang Zhang

**Affiliations:** 1 Guangdong Provincial Key Laboratory of Agro-Animal Genomics and Molecular Breeding, Guangdong Provincial Key Laboratory of Animal Nutrition Control, South China Agricultural University, 483 Wushan Road, Guangzhou, 510642 China; 2 Institute of Animal Science and Veterinary Medicine, Hainan Academy of Agricultural Sciences, Haikou, 571100 China

**Keywords:** Biochemistry, Biotechnology, Molecular biology, Diseases, Health care

## Abstract

Obese individuals are more susceptible to comorbidities than individuals of healthy weight, including cardiovascular disease and metabolic disorders. MicroRNAs are a class of small and noncoding RNAs that are implicated in the regulation of chronic human diseases. We previously reported that miR-125b plays a critical role in adipogenesis in vitro. However, the involvement of miR-125b-2 in fat metabolism in vivo remains unknown. In the present study, miR-125b-2 knockout mice were generated using CRISPR/CAS9 technology, resulting in mice with a 7 bp deletion in the seed sequence of miR-125b-2. MiR-125b-2 knockout increased the weight of liver tissue, epididymal white fat and inguinal white fat. MiR-125b-2 knockout also increased adipocyte volume in HFD-induced obese mice, while there were no significant differences in body weight and feed intake versus mice fed a normal diet. Additionally, qRT-PCR and western blot analysis revealed that the expression of the miR-125b-2 target gene SCD-1 and fat synthesis-associated genes, such as PPARγ and C/EBPα, were significantly up-regulated in miR-125b-2KO mice (*P* < 0.05). Moreover, miR-125b-2KO altered HFD-induced changes in glucose tolerance and insulin resistance. In conclusion, we show that miR-125b-2 is a novel potential target for regulating fat accumulation, and also a candidate target to develop novel treatment strategies for obesity and diabetes.

## Introduction

Obesity is rapidly rising as a public health concern all over the world. Being on the overweight side of the weight continuum is associated with higher risk of serious chronic diseases^1^, including type 2 diabetes mellitus, non-alcoholic fatty liver disease, hypertension, and several cancers^2^. The causes of obesity are related to diet, exercise and genetic factors, but the most direct cause is an increase in the number or volume of fat cells. The number of adipocytes is largely related to processes of adipocyte differentiation^3,4^, and it is therefore of great importance to elucidate the molecular mechanisms of adipocyte differentiation in order to better prevent and treat of obesity and obesity-related metabolic diseases.

MicroRNAs (miRNAs) are a class of non-coding RNAs that post-transcriptionally repress expression or translation of target mRNAs through sequence-specific regulation of gene expression^5^, and are known to regulate thousands of gene targets^6^. A large number of miRNA markers have been identified in many chronic diseases, including cancer^7^, cardiovascular disease, and type 2 diabetes^8^. It has recently been shown that obesity in humans is directly related to abnormal miRNA expression. For instance, miR-16-5p^9^, miR-103^10^, and miR-26b^11^positively regulate adipogenesis, while miR-27a/b^12,13^, miR-130^14^, and miR-93^15^ inhibit adipogenesis.

MiR-125b is located at the chromosome 11q23 and 21q21 loci, and the mature miR-125b sequences are highly evolutionarily conserved among pigs, humans, and mice. Mature miR-125b originates from two precursors, miR-125b-1 and miR-125b-2. Members of the miR-125b family exhibit disease-suppressing properties in a number of disease contexts^16^. Additionally, miR-125b has many functions, including inhibiting osteoblastic differentiation^17^, inhibiting metastasis of hepatocellular carcinoma^18^, and regulating cancer cell proliferation and lipogenesis^19,20^ by regulating expression of its target genes^21^. In 3T3 preadipocyte cells, miR-125b promotes murine white adipocyte differentiation, likely through a mechanism involving SMAD4 inhibition^22^. MiR-125b may also serve as a regulator of human adipocyte differentiation^19^, as its expression levels are negatively associated with UCP1 mRNA expression in both white and brown adipocytes derived from mice and humans^23^. Upregulation of miR-125b by oestrogen protected against NAFLD in female mice. The expression of miR-125b has also been associated with obesity in mice and humans. Furthermore, MiR-125b was shown to be downregulated in adipose tissue from high fat diet-induced obese mice^24^.

In general, the expression levels of miRNAs are negatively related to the expression levels of their target genes.It has been previously reported that miR-125b-2 expression is reduced in the adipose tissue of wild type (WT) mice fed a HFD. Consumption of a HFD can increase the expression and enzyme activity of SCD-1in rat adipose tissue^25–27^. We previously demonstrated that miR-125b-2 inhibits lipogenesis by targeting SCD-1 in vitro^28^. Nevertheless, the function of miR-125b-2 in adipogenesis in vivo has not been elucidated. Therefore, the purpose of the present study was to determine the effects of miR-125b-2 on fat accumulation in vivo using a miR-125b-2 knockout mouse model.

## Results

### Generation and validation of miR-125b-2 knockout (KO) mice

FVB mice were purchased from Beijing Weitong Lihua Experimental Animal Technology Co. Ltd. (permission number: SCXK (JING) 2012–0001). Global miR-125b-2 knockout mice (miR-125b-2KO mice; FVB) were generated by Cyagen Biosciences (Guangzhou, China) using CRISPR/CAS9 methodology, resulting in a 7 bp deletion in the seed sequence of the miR-125b-2 gene in the miR-125b-2KO mice (Supplemental Fig. 1,A); the deletion was confirmed by sequencing. Mice of the miR-125b-2KO F1 generation were mated with WT mice to obtain offspring that were WT, heterozygous for miR-125b-2KO, and homozygous for miR-125b-2KO. The genotype of the offspring was confirmed by tail genotyping. Briefly, the tip of the tail was removed from the offspring (according to IACUC approved methods), DNA was extracted, and DNA integrity confirmed by electrophoresis. The isolated DNA was used as template for PCR amplification using custom-designed miR-125b-2 primers. The PCR products were sent to Sangon Biotech (Shanghai) Co, Ltd for sequencing. The sequencing results were compared using Chromas software and NCBI-BLAST to confirm the genotype of miR-125b-2KO homozygous mice (Supplemental Fig. 1B, C). In order to verify that the miR-125b-2KO mice exhibited global knockout, 6-week-old miR-125b-2KO and WT male mice were euthanized (n = 8), and samples of heart, liver, spleen, lung, kidney, leg muscle, epididymal fat (EpiWAT), and inguinal fat (IngWAT) were collected for RNA extraction. After RNA extraction, the expression of miR-125b-2 in the different tissue samples was were measured by qRT-PCR. The expression of miR-125b-2 in all tissue samples from miR-125b-2KO mice was significantly lower than in WT mice (Supplemental Fig. 1D). These data demonstrate that miR-125b-2 was knocked out globally in the miR-125b-2KO mice. Finally, the miR-125b-2KO mice were bred together, and the resulting homozygous miR-125b-2KO offspring were used for subsequent experiments.

**Figure 1:**
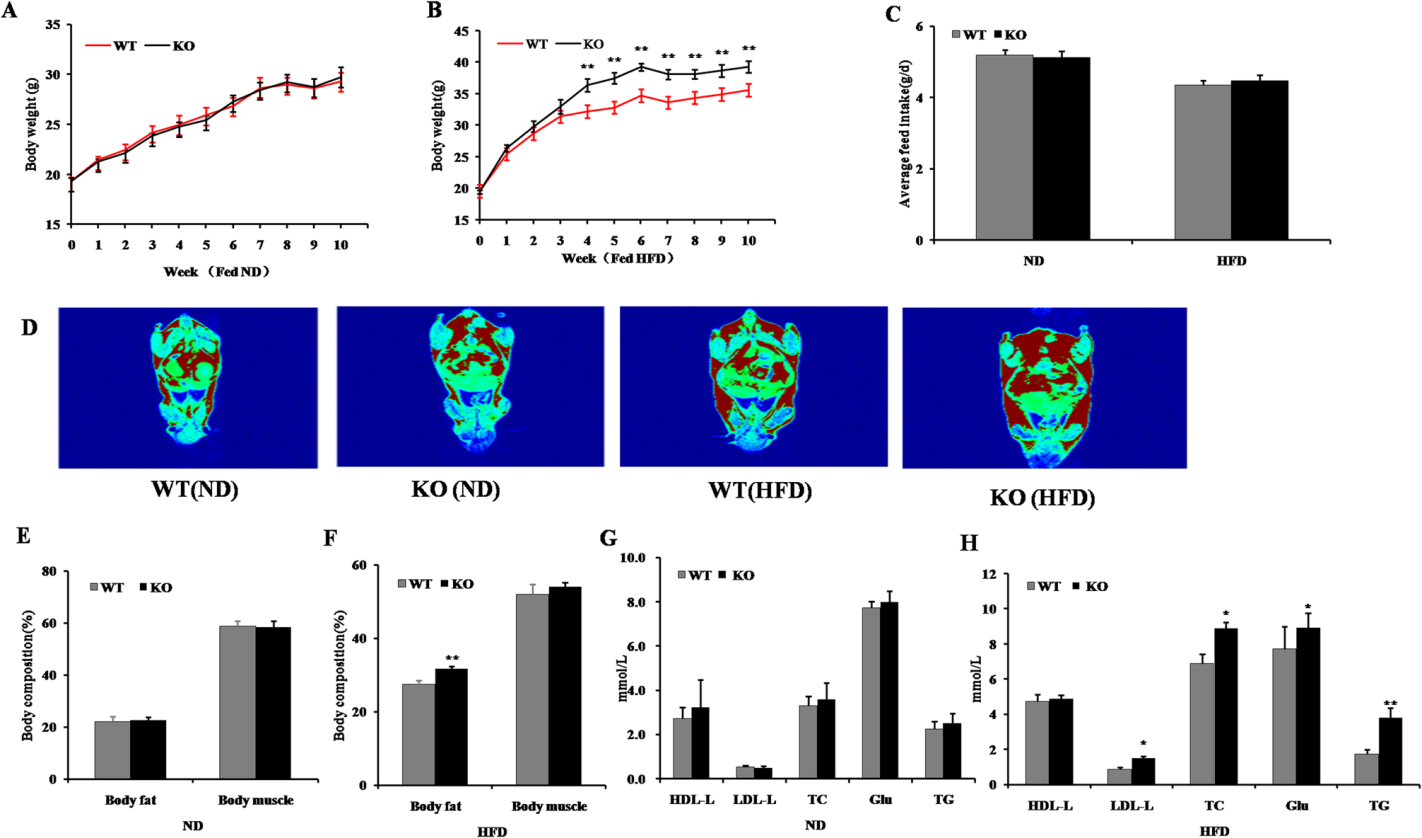
MiR-125b-2 knockout increased HFD-induced obesity**.** (**A**) The body weight (BW) between WT and KO mice fed a ND was not significantly different (*P* > 0.05). (**B**) MiR-125b-2 knockout promotes HFD-induced obesity in male mice. (**C**) Feed intake between WT and KO mice fed ND and HFD. (**D**) Body imaging results of body fat distribution in WT and KO mice fed ND or HFD. (**E**) The body fat and body muscle content in WT and KO mice in the ND group. (**F**) The body fat and body muscle content in WT and KO mice in the HFD group. (**G**) The serum HDL-L, LDL-L, TC, GLU, and TG contents of WT and KO mice fed ND or (**H**) HFD. Data are presented as mean ± SE (*P < 0.05, **P < 0.01*,* n = 8 mice per group).

### MiR-125b-2KO increases HFD-induced obesity in mice

WT and miR-125b-2KO mice were fed normal diet (ND) or high fat diet (HFD) for ten weeks. Compared with WT mice, miR-125b-2KO mice exhibited significantly greater body weight increase when fed a HFD (*P* < 0.05; Fig. 1B), and the body weight of miR-125b-2KO mice fed a HFD was 10.50% greater than the body weight of WT HFD mice (*P* < 0.05; Fig. 1B); in contrast, miR-125b-2KO mice fed a ND did not exhibit increased body weight gain compared to WT mice fed ND (Fig. 1A). The feed intakes were not significantly different between WT and miR-125b-2KO mice fed either kind of diet (*P* > 0.05; Fig. 1C). Body imaging revealed that miR-125b-2KO mice fed a HFD had significantly increased body fat content compared to WT mice fed a HFD, but this difference was not seen among mice fed a ND (Fig. 1D). Further body composition analysis revealed that body fat and muscle content did not differ between WT and miR-125b-2KO mice in the ND group (*P* > 0.05) (Fig. 1E), but the body fat content of miR-125b-2KO mice fed a HFD was significantly higher than that of WT mice fed a HFD (*P* < 0.05, Fig. 1F), and the body fat content of miR-125b-2KO mice was ~ 15.00% greater than that of WT mice.. The serum high density lipoprotein cholesterol (HDL-L), low density lipoprotein cholesterol (LDL-L), total cholesterol (TC), glucose (GLU) and triglyceride (TG) levels remained unchanged in the WT and miR-125b-2KO mice fed a ND (*P* > 0.05; Fig. 1G); however, low density lipoprotein cholesterol (LDL-L), total cholesterol (TC), glucose (GLU) and triglyceride (TG) levels were significantly increased in miR-125b-2KO mice fed a HFD relative to WT mice fed a HFD (*P* < 0.05; Fig. 1H). In particular, the triglyceride content of miR-125b-2KO mice was ~ 60% higher than that of WT mice.

### MiR-125b-2KO increases adipogenesis in HFD-induced obese mice

**Figure 2:**
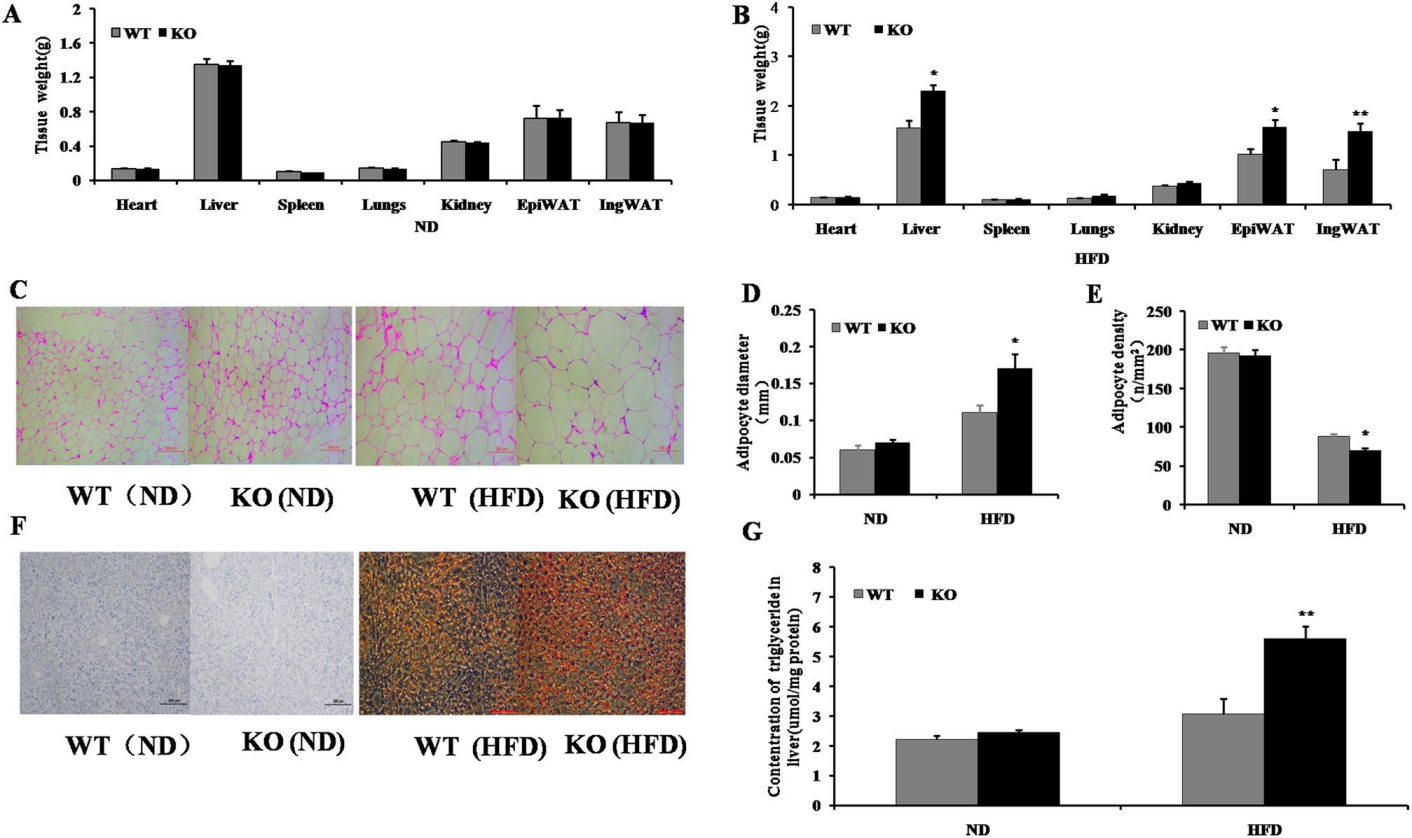
MiR-125b-2 knockout increased the weight of liver, EpiWAT, and IngWAT tissue, and increased adipocyte volume and diameter in HFD-induced obese mice. (**A**) The weight of tissues and organs from WT and KO mice fed ND or (**B**) HFD. (**C**) IngWAT adipocyte volume, (**D**) diameter, and (**E**) adipocyte density in WT and KO mice fed ND or HFD. (**F**) Lipid droplet number and (**G**) Liver triglycleride contents in WT and KO mice fed ND or HFD. Data are presented as mean ± SE (*P < 0.05, **P < 0.01.) Microscope images C and F are representative images, Scale bar: 100 × magnification, 200 μm; n = 24 photos per animal / per group, n = 8 mice per group.

There was no significant difference in the weight of tissues and organs between WT and miR-125b-2KO mice fed a normal diet for ten weeks (*P* > 0.05; Fig. 2A). Interestingly, after being fed a HFD for ten weeks, the weight of liver, epididymal white fat (*P* < 0.05), and inguinal white fat (*P* < 0.01) in the miR-125b-2KO mice were significantly increased relative to the WT mice. Notably, the inguinal white fat was increased the most, and was about twice as much in miR-125b-2KO mice fed HFD than WT mice fed HFD. However, the weight of other tissues and organs were not significantly different (*P* > 0.05; Fig. 2B). Further histological and immunohistochemical examination showed that the volume and diameter of inguinal white fat adipocytes were significantly increased (*P* < 0.05;Fig. 2C,D), and the density of inguinal white fat adipocytes was significantly decreased (*P* < 0.05; Fig. 2E). Moreover, the lipid droplet number in liver tissue was also significantly increased (*P* < 0.05; Fig. 2F), and the triglyceride content in liver tissue of miR-125b-2KO mice was about 80% greater than that of WT mice (*P* < 0.05; Fig. 2G). However, there were no significant differences in inguinal fat adipocyte volume and diameter, liver lipid droplet number, and triglyceride content between WT and miR-125b-2KO mice in the normal diet group (*P* > 0.05; Fig. 2C–G). These results indicate that miR-125b-2 knockout can promote lipogenesis in mice fed a HFD.

### MiR-125b-2KO decreases insulin sensitivity in HFD-induced obese mice

**Figure 3:**
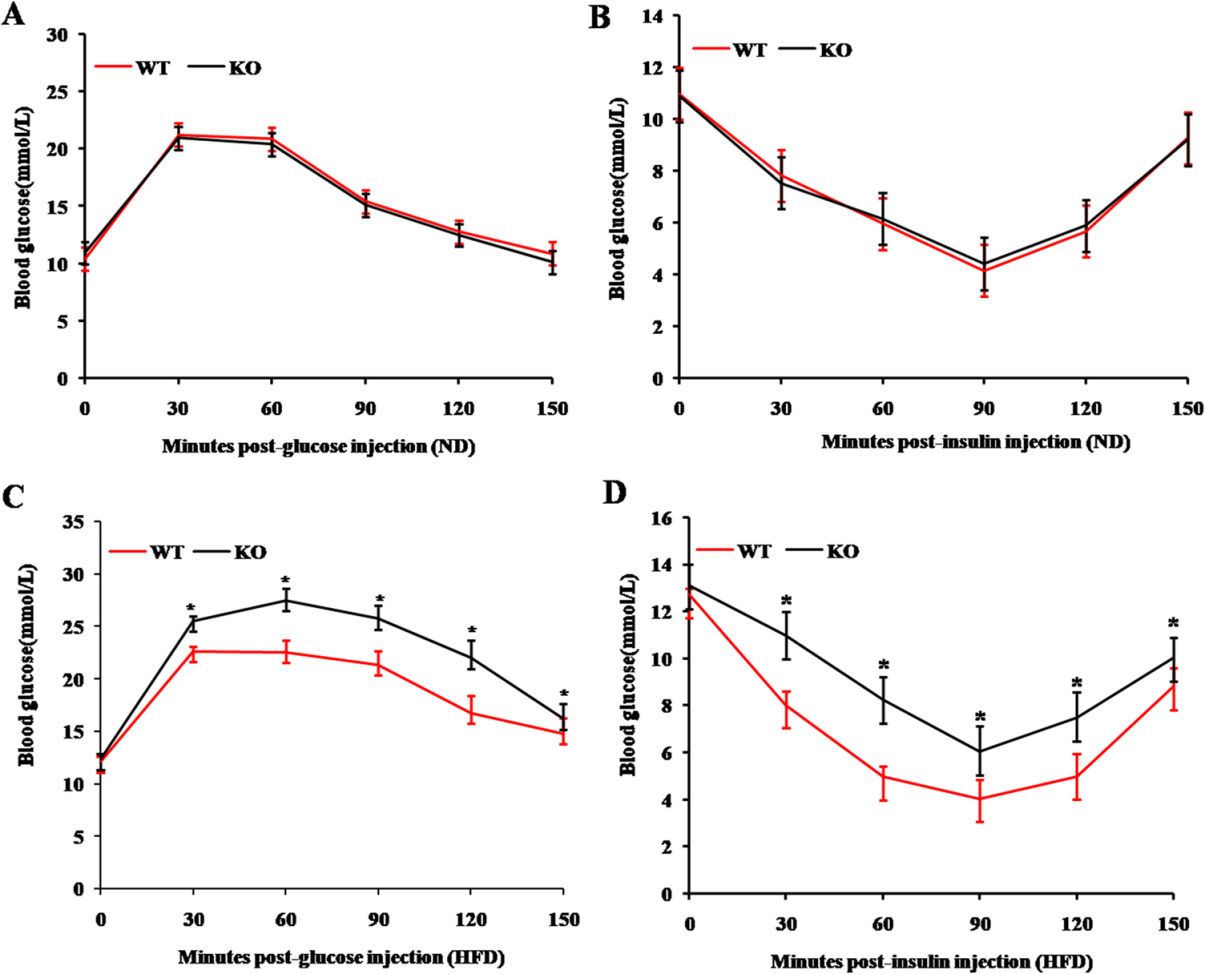
MiR-125b-2 knockout decreased insulin sensitivity in HFD induced obese mice. (**A**) GTT and (**B**) ITT were performed after feeding ND for 10 weeks; or (**C**) GTT and (**D**) ITT after feeding HFD for 10 weeks. GTT = glucose tolerance test; ITT = insulin tolerance test. Data are presented as mean ± SE (*P < 0.05, n = 8 mice per group).

Obesity is associated with a reduced ability to metabolize glucose and with an increased risk of type 2 diabetes. Therefore, glucose tolerance tests were conducted to examine serum glucose levels after injection of glucose or insulin. There were no significant differences in glucose tolerance or insulin resistance between WT mice and miR-125b-2KO mice fed a normal diet (*P* > 0.05; Fig. 3A,B). However, when fed a HFD, miR-125b-2KO mice recovered serum glucose levels more slowly than WT mice following injection with glucose or insulin (Fig. 3C,D). These results indicate that miR-125b-2 KO reduces glucose utilization and insulin sensitivity in mice fed a HFD.

### MiR-125b-2KO increases expression of genes related to fat synthesis

**Figure 4:**
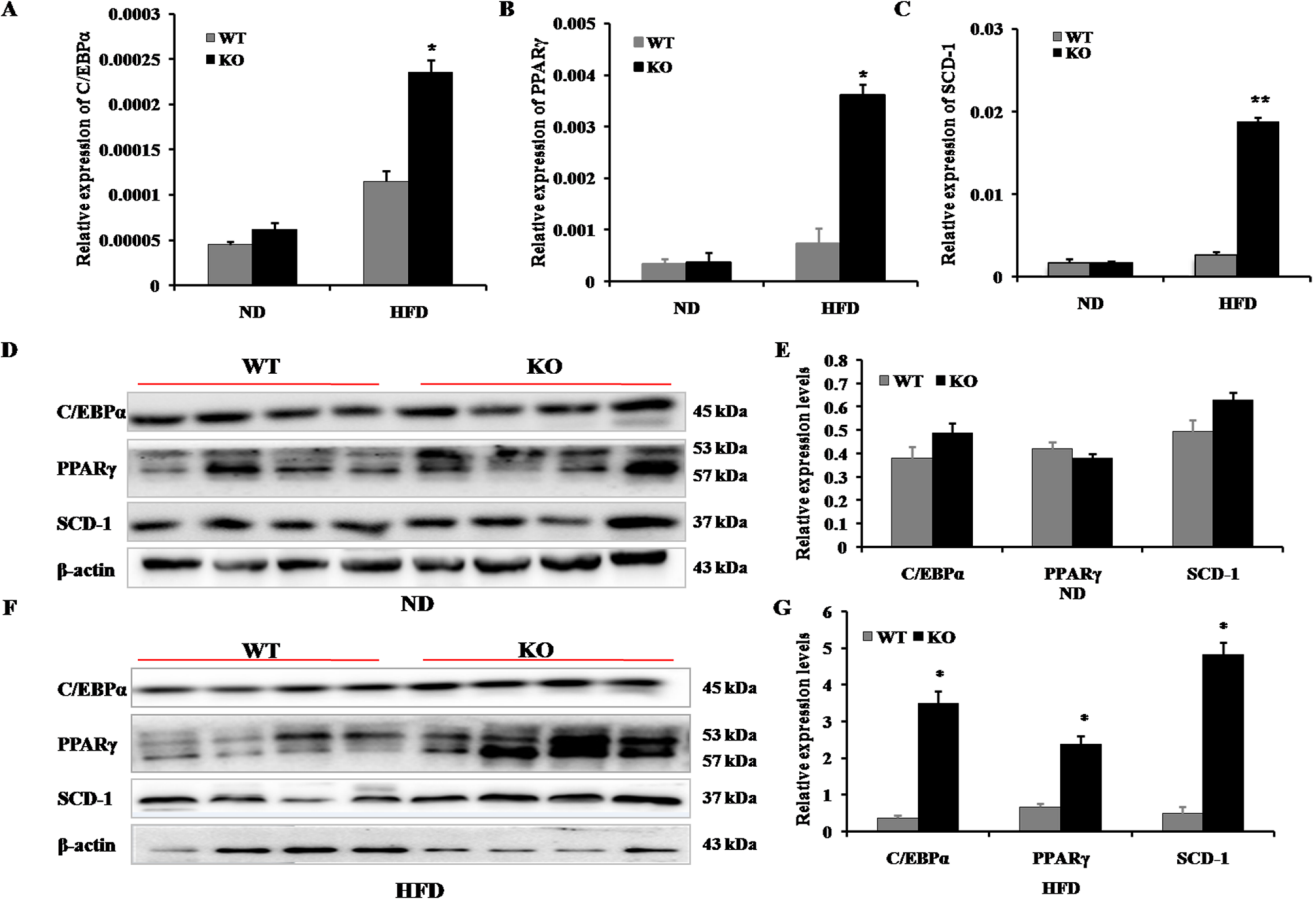
MiR-125b-2 increased expression levels of fat synthesis-associated genes. (**A**–**C**) Expression levels of C/EBPα, PPARγ, and SCD-1 mRNA in WT and KO mice fed a ND or HFD. SCD-1, PPARγ, and C/EBPα were significantly increased in KO mice fed a HFD. (**D**,**E**) Expression levels of C/EBPα, PPARγ and SCD-1 protein in WT and KO mice fed a ND. (**F**,**G**) Expression levels of C/EBPα, PPARγ and SCD-1 protein in WT and KO mice fed a HFD. C/EBPα, PPARγ, and SCD-1 protein expression was significantly increased in KO mice fed a HFD. The images displayed in Figures D and F are cropped blots, the full-length blots of (**D**,**F**) are presented in Supplementary Fig. 2. Data are presented as mean ± SE (*P < 0.05, ** *P* < 0.01, n = 8 mice per group).

Our previous results show that miR-125b-2KO mice with HFD-induced obesity were more obese than WT mice fed a HFD. Therefore, we evaluated the expression levels of fat synthesis-related genes in IngWAT. qRT-PCR revealed that miR-125b-2KO significantly up-regulated the mRNA levels of SCD-1 (*P* < 0.01), PPARγ, and C/EBPα, in mice fed a HFD (*P* < 0.05; Fig. 4A–C). Western blot analysis further verified up-regulation of SCD-1, C/EBPα, and PPARγ (Fig. 4F,G). However, there were no significant differences in expression of these genes between WT and miR-125b-2KO mice fed a ND (Fig. 4D,E).

## Discussion

The increasing incidence of obesity is concerning for public health, as obesity can lead to an increase in the incidence of many diseases, including as type 2 diabetes^29^, cardiovascular disease^30^, and arteriosclerosis^31^. MicroRNAs can influence many biological functions, including metabolism^32^ and adipose tissue development and function^33^. For example, a recent study reported that miR-143 effectively inhibited adipocyte differentiation^34^. Another study demonstrated that microRNA-221 is up-regulated in obesity and affects fat metabolism downstream of leptin and TNFα^35^. The members of the miR-125 family are located on different chromosomes and play important roles in the occurrence and development of human diseases^36^. In the past few years, remarkable advances have been made in the study of miR-125b. MiR-125b has been found to be involved in human breast cancer^37^, thyroid cancer^38^, hepatocellular carcinoma^18^, oral cancer^39^, colorectal cancer^40^, bladder cancer^41^, prostate cancer^42^, osteosarcoma^43^, and lung cancer^44^. MiR-125b is upregulated or downregulated in different cancers under pathophysiological conditions^45^. MiR-125b has also been reported to regulate lipogenesis in mouse, human and pig cells in vitro^19,22,28^. However, up to now, there has been no report on the involvement of miR-125b-2 in adipogenesis in vivo.

The clustered regularly interspaced short palindromic repeat (CRISPR)/CRISPR-associated protein (Cas) system, is a highly efficient genome-editing technique that has been widely used in various fields of life science research. CRISPR/Cas-mediated genome editing has achieved such revolutionary progress due to the advantages of a highly efficient mutation rate and a simple system for designing of target-specific sgRNA to guide the genome editing^46,47^. CRISPR/Cas-mediated in vivo genome editing has been used in the production of knockout mice^48–50^. However, there are still some drawbacks to this technique, and approximately one-third of the homozygous gene knockout efforts results in embryonic lethality or birth mortality in mice. In addition, CRISPR/Cas-mediated gene editing may cause other genes to compensate for the knockout of any given gene, leaving no significant phenotypic changes after gene knockout^51^.

In the present study, we did not find any significant differences in body weight and feed intake between miR-125b-2KO mice and WT mice fed with a normal diet. Interestingly, the body weight and body fat content of miR-125b-2KO mice fed a high fat diet were significantly increased compared to WT mice fed a high fat diet. Furthermore, the serum LDL-L, TC, GLU, and TG content of miR-125b-2KO mice fed a high fat diet were also significantly increased compared to WT mice fed a high fat diet. Our findings are in agreement with previous research results^52,53^. We speculate that miR-125b-2 may not exhibit any obvious phenotype under standard controlled laboratory conditions, like other mouse strains lacking specific miRNAs^52^, but the phenotype(s) may become more readily apparent under conditions of stress, such as in response to excessive calorie intake^54^. The reason is possibly that miR-125b-2 has numerous targets, and although the repressive influence of miR125b-2 in adipose tissue likely contributes to lipid metabolism, the combined functions of the miRNA in multiple tissues and on multiple targets are undoubtedly involved in mediating the knockout phenotype.

Since feed intake did not change, we speculate that the increased obesity induced by HFD in the miR-125b-2KO mice is likely caused by changes in energy metabolism. These results suggest that the body weight gain of miR-125b-2KO mice fed a HFD was likely due to increased lipogenesis, indicating that miR-125b-2 may play an important role in regulating lipogenesis in vivo. Obesity is often associated with increased fat weight, increased adipocyte size^55^, and fatty liver disease^56^. In the present study, the weight of EpiWAT and IngWAT, and IngWAT cell volume and size of miR-125b-2KO mice fed a HFD were significantly increased compared to WT mice fed a HFD. Moreover, the liver weight, lipid droplets accumulation, and triglyceride content were also increased in miR-125b-2KO mice fed a HFD, compared to WT mice fed a HFD. The results above provide further evidence that miR-125b-2 is a crucial factor in regulating adipogenesis.

Concurrent with increasing obesity worldwide is an increase in various metabolic diseases, including type 2 diabetes, and it is important to elucidate mechanisms of insulin resistance to identify new targets for intervention and treatment of obesity-related insulin resistance^57^. MiRNAs are known to play important roles in regulating insulin resistance. For example, down-regulation of miR-543 alleviates insulin resistance by targeting the SIRT1^58^, miR-27a induces insulin resistance by repressing PPARγ^59^, and miR-27b and miR-130a were linked to adipocyte insulin resistance by targeting the insulin receptor itself^12,14,60^. Adipose tissue is not only an organ for storing fat and energy, but also an important endocrine organ^61^. We found that, compared to WT mice fed a HFD, miR-125b-2KO mice fed a HFD had significantly slower glucose recovery after injection with glucose or insulin, indicating that miR-125b-2 plays a role in glucose tolerance and insulin sensitivity in vivo.

The crucial role of SCD-1 in regulating lipid metabolism has been well documented^62^. Recent data suggest that SCD-1 is regulated by a number of miRNAs. For example, overexpressed miR-27a reduced lipid accumulation in primary hepatocytes and in mouse liver tissue by repressing expression levels of SCD-1^63^. The regulation of BM-MSCs of patients with type 2 diabetes mellitus (T2DM) by SCD-1 is a necessary condition for osteogenesis mediated through the miR-203a/FOS and miR-1908/EXO1 regulatory pathways^64^. MiR-600 acts as a regulator of WNT signalling through SCD-1 to regulate breast tumour genesis^65^. Differentially methylated regions (DMR) in the promoter region of miR-4335 can affect fatty acid composition by targeting SCD-1^66^. In addition, as a rate-limiting enzyme in fat synthesis, SCD-1 plays an important role in driving susceptibility to diet-induced obesity^67,68^. SCD-1 deficiency decreases body weight, white adipose tissue mass, and hepatic TG in high-fat diet-fed obese mice^68,69^. It was previously found that HFD increases SCD-1 expression and enzyme activity levels in both rat liver and adipose tissue^27,70,71^. By contrast, the expression of miR-125b-2 was downregulated in adipose tissue from high fat diet-induced obese mice and overweight humans^24,72^. In our former study, we found that miR-125b was significantly down-regulated in the adipose tissue of fat-rich pigs (Lantang, a local breed in China) compared with fat-less (Landrace) pigst^73^. In vitro, we validated SCD-1 as a target of miR-125b using a dual-luciferase assay, and we found that miR-125b could inhibit SCD-1 expression by targeting the 3′-UTR of SCD-1 mRNA, which affected lipogenesis and fat composition of porcine adipocytes. Moreover, in vivo experiments in mice showed that injection of a miR-125b expression vector decreased the concentration of hepatic triglycerides and reduced SCD-1 protein content compared to a control group. In the present study, we found that SCD-1 was significantly upregulated in IngWAT of miR-125b-2KO mice compared to WT mice fed a HFD. These results above further confirm the role of miR-125b-2 in regulating fat accumulation in animals fed a HFD by targeting SCD-1^74,75^.

## Conclusions

In conclusion, we found that miR-125b-2KO mice fed a HFD accumulated more fat than WT mice fed HFD. Moreover, miR-125b-2 knockout resulted in reduced glucose utilization and insulin sensitivity in mice fed a HFD. Our study is the first to demonstrate that miR-125b-2 plays a crucial role in regulating lipogenesis and insulin resistance in vivo, especially in the context of a high fat diet. Our results indicate that miR-125b-2 is a potential novel target for the treatment of obesity and obesity-associated metabolic disorders. The ability to modulate miR-125b-2 activity through the systemic delivery of its inhibitors or mimics without toxicity provides chances for intervening in obesity-related diseases. Moreover, miR-125b-2 can be used as a biomarker of obesity and obesity-related complications, and may provide new strategies for the diagnosis and treatment of obesity-related diseases.

## Materials and methods

### Ethics statement

The animal experiments were handled in strict compliance with the guidelines of Guangdong Province on the Review of Welfare and Ethics of Laboratory Animals approved by the Guangdong Province Administration Office of Laboratory Animals. All procedures were conducted according to the protocol (SCAU-AEC-2016–0714, 14 July 2016) and approved by the Animal Ethics Committee of South China Agricultural University. All experiments were conducted in accordance with “The Instructive Notions with Respect to Caring for Laboratory Animals” issued by the Ministry of Science and Technology of the People’s Republic of China.

### Animal experiments and sample acquisition

A total of 32 weaned male FVB mice (16 WT and 16 miR-125b-2KO mice) were acquired from the experimental field of the animal observation station in South China Agricultural University. Mice were allowed to acclimate for one week prior to the experimental period. Mice were maintained under a regular 12 h light and 12 h dark schedule at a temperature of 25 °C ± 2 °C and a relative humidity of 70% ± 10% throughout the experimental period. The mice had an initial body weight (BW) of 19.36 ± 0.46 g. Mice were randomly assigned to four groups with eight animals per group; 16 mice (8 WT and 8 miR-125b-2KO mice) were fed normal diet (ND, n = 8 per group) and 16 mice (8 WT and 8 miR-125b-2KO mice) were fed high-fat diet (HFD, 60% of total calories from fat, n = 8 animals per group). The experiment lasted for 10 weeks. Body weight was checked weekly. Feed intake during the entire test period was recorded and quantified. At the end of the trial, blood samples were collected from each mouse. All mice were euthanized by cervical dislocation, and sterilized scissors and tweezers were used to open the skin and remove IngWAT. Then the abdomen was opened and tissue samples of EpiWAT, heart, liver, spleen, lung, and kidney were collected. The weight of visceral organs, epididymal fat (EpiWAT), and inguinal fat (IngWAT) were recorded. All samples were stored at − 80 °C for further evaluation. The experimental diets were purchased from the Guangdong Laboratory Animal Center. The dietary composition and nutritional levels are shown in Supplemental Table 1.

### Blood biochemical indexes and liver triglyceride content

Blood biochemical indexes and liver triglyceride content were measured using a commercial kit, according to the manufacturer’s instructions (NanJingJianCheng Bioengineering Institute, NanJing, China). Glucose (GLU) was determined using a glucose oxidase assay kit (Serial number: F006-1-1). Total cholesterol (TC) and triglyceride (TG) content were determined by a Single reagent GPO-PAP assay kit (Serial number: A111-1-1, A110-1–1). High-density lipoprotein cholesterol (HDL-C) and low-density lipoprotein cholesterol (LDL-C) were determined by a Direct double reagent assay kit (Serial number: A112-1-1,A113-1-1).

### Body imaging and body composition assay

At the end of the experiment, 5% chloral hydrate anesthetic was prepared and mice were injected with 1.5 ml intraperitoneally. Body imaging and body composition were determined using quantitative magnetic resonance (n = 8, QMR, Niumag Corporation, Shanghai, China). Images and data were saved and recorded for further analysis and statistics.

### Histological analysis

Fat tissues (inguinal fat) were fixed in 10% formaldehyde for 60 min at room temperature, then were embedded in paraffin for 1 h and cut into 5 µm slices. Sections were deparaffinized and rehydrated using xylene, ethanol, and water by standard procedures. Sections were stained with hematoxylin/eosin. At the same time, we also analyzed and compared the diameter and density of inguinal fat between the WT and miR-125b-2KO mice fed with the ND or HFD. The adipocyte diameter was calculated as follows: three slices were made for each mouse in each group, and 100-fold fields were randomly selected for photography in each slice. When taking pictures, the entire field of view should be as full of tissue as possible, to ensure that the background light in each photo is consistent. Image-pro Plus 6.0 software was used to measure the diameter (mm) of 5 fat cells in each slice, and the average value of each group was calculated (n = 24 photos per animal/per group). The adipocyte density (n/ mm^2^) = adipocyte number/ field area (mm^2^).

Oil Red O staining in liver tissue was carried out as described in our previous report^25^. Briefly, liver tissues were frozen in liquid nitrogen, and then cut into 5 µm sections using a cryostat. Slices were washed three times with water, fixed in 4% formaldehyde for 30 min, washed again with water three times, and stained with Oil Red O (Sigma-Aldrich, Shanghai, China) for 1 h. The stained slices were washed three times with water and then photographed.

### Glucose and insulin tolerance tests

Mice fasted for 16 h prior to the glucose tolerance test, or for 4 h prior to the insulin tolerance test. For the male WT/miR-125b-2KO experiments, glucose tolerance was tested at 10 weeks post-ND/HFD, and for the chimera experiment, glucose tolerance was tested at 9 weeks after ND-HFD and insulin tolerance at 10 weeks post-ND/HFD. Body weight pre-fasting and post-fasting was recorded. The mice were injected intraperitoneally with glucose (1.5 g/kg) or insulin (0.75 U/kg). Animals receiving both glucose and insulin tolerance tests had > 7 day between tests to minimize stress. Tail blood was collected at 0, 30, 60, 90, 120 and 150 min post-glucose injection. Blood glucose levels were immediately measured using a glucometer (Accu-Check Active, Roche, Germany).

### RNA analysis and qRT-PCR

Total RNA was extracted from tissues using Trizol reagent (Invitrogen), and RNA concentration was detected by a spectrophotometer (Nanodrop 2000, Thermo Fisher). Total RNA (2 µg) was reverse-transcribed into cDNA using the MLV reverse transcriptase kit (Promega) with OligodT18. Real-time PCR was carried out in a STRATAGENE Mx3005P sequence detection system with SYBR Green Master Mix (Promega). The mRNA expression level for each gene was normalized to the expression of β-actin in each sample. All primers are shown in Supplemental Table 2.

### MiR-125b-2 qRT-PCR detection

Expression of miR-125b-2 was performed using stem-loop qRT-PCR, as previously described^76^. Two looped antisense primers of miR-125b-2 (5′-ACACTCCAGCTGGGTCCCTGAGACCCTAAC-3′) were used for reverse transcription. The qPCR forward primer sequences for miR-125b-2 are 5′- TGGTGTCGTGGAGTCG-3′. The expression of miRNA was normalized to the level of the U6, primers are shown in Supplemental Table 2. The 2^−∆Ct^ method was used to analyze the relative expression miRNA expression.

### Protein extraction and western blot

Protein was extracted from inguinal fat using radio immunoprecipitation assay (RIPA) buffer. The protein concentration was evaluated using the BCA protein assay (ThermoFisher). Briefly, 20 µg total protein was separated on a 10% SDS-PAGE gel. After electrophoresis, total proteins were transferred onto a PVDF membrane, and the PVDF membranes were cut according to the molecular weight of the target protein. The PVDF membranes were blocked with 5% skimmed milk and incubated overnight at 4 °C with primary antibodies against anti-SCD-1, PPARγ (Cell Signaling Technology, Inc.), and C/EBPα (Santa Cruz Biotechnology, Inc). β-Actin protein expression was evaluated as a normalization control (Abcam, Cambridge, UK). After incubation with primary antibodies, anti-mouse IgG secondary antibodies (Cell Signaling Technology) were added to the membranes and incubated for 60 min at room temperature. The blot was scanned in a Fluor ChemM instrument (ProteinSimple, Santa Clara, California, USA). The data were analyzed by Image J software and expressed as fold-change relative to the control group after normalizing against β-actin.

### Statistics analysis

All data are expressed as the mean ± standard error of the mean (SEM). The significance of differences between groups was determined using t-test for comparison of 2 groups, and one-way analysis of variance (ANOVA) with post hoc test of least significant difference (LSD) test for multiple comparisons. Statistical analyses were performed using SPSS 20.0 software. Differences were considered statistically significant at *P* < 0.05.

## Supplementary information

Supplementary Information.

## References

[1] Williams EP, Mesidor M, Winters K, Dubbert PM, Wyatt SB (2015). Overweight and obesity: prevalence, consequences, and causes of a growing public health problem. Current Obes. Rep..

[2] Bluher M (2019). Obesity: global epidemiology and pathogenesis. Nat. Rev. Endocrinol..

[3] Katsogiannos P (2019). Early changes in adipose tissue morphology, gene expression, and metabolism after RYGB in patients with obesity and T2D. J. Clin. Endocrinol. Metab..

[4] Mclaughlin T (2016). Adipose cell size and regional fat deposition as predictors of metabolic response to overfeeding in insulin-resistant and insulin-sensitive humans. Diabetes.

[5] Yamamura S, Imaisumida M, Tanaka Y, Dahiya R (2018). Interaction and cross-talk between non-coding RNAs. Cell. Mol. Life Sci..

[6] Lagosquintana M, Rauhut R, Lendeckel W, Tuschl T (2001). Identification of novel genes coding for small expressed RNAs. Science.

[7] Lu J (2005). MicroRNA expression profiles classify human cancers. Nature.

[8] Dalessandra Y (2010). Circulating microRNAs are new and sensitive biomarkers of myocardial infarction. Eur. Heart J..

[9] Xu J, Zhang L, Shu G, Wang B (2019). microRNA-16–5p promotes 3T3-L1 adipocyte differentiation through regulating EPT1. Biochem. Biophys. Res. Commun..

[10] Trajkovski M (2011). MicroRNAs 103 and 107 regulate insulin sensitivity. Nature.

[11] Song G (2014). The role of microRNA-26b in human adipocyte differentiation and proliferation. Gene.

[12] Kim SY (2010). miR-27a is a negative regulator of adipocyte differentiation via suppressing PPARgamma expression. Biochem. Biophys. Res. Commun..

[13] Hsu C, Lai C, Lin C, Yeh K, Her GM (2017). MicroRNA-27b depletion enhances endotrophic and intravascular lipid accumulation and induces adipocyte hyperplasia in zebrafish. Int. J. Mol. Sci..

[14] Lee EK (2011). miR-130 Suppresses Adipogenesis by Inhibiting Peroxisome Proliferator-Activated Receptor Expression. Mol. Cell. Biol..

[15] Cioffi M (2015). MiR-93 controls adiposity via inhibition of Sirt7 and Tbx3. Cell Rep..

[16] Sun Y, Lin K, Chen Y (2013). Diverse functions of miR-125 family in different cell contexts. Journal of Hematology & Oncology.

[17] Mizuno Y (2008). miR-125b inhibits osteoblastic differentiation by down-regulation of cell proliferation. Biochem. Biophys. Res. Commun..

[18] Jia H (2012). MicroRNA-125b functions as a tumor suppressor in hepatocellular carcinoma cells. Int. J. Mol. Sci..

[19] Rockstroh D, LöFfler D, Kiess W, Landgraf K, KöRner A (2016). Regulation of human adipogenesis by miR125b-5p. Adipocyte.

[20] Giroud M (2016). miR-125b affects mitochondrial biogenesis and impairs brite adipocyte formation and function. Mol. Metab..

[21] Huang L (2011). MicroRNA-125b suppresses the development of bladder cancer by targeting E2F3. Int. J. Cancer.

[22] Ouyang D (2015). MicroRNA-125b-5p inhibits proliferation and promotes adipogenic differentiation in 3T3-L1 preadipocytes. Acta Biochim. Biophys. Sin..

[23] Maude (2016). miR-125b affects mitochondrial biogenesis and impairs brite adipocyte formation and function. Mol. Metab..

[24] Xie H, Lim B, Lodish HF (2009). MicroRNAs induced during adipogenesis that accelerate fat cell development are downregulated in obesity. Diabetes.

[25] Cedernaes J, Alsiö J, Västermark A, Risérus U, Schiöth HB (2013). Adipose tissue stearoyl-CoA desaturase 1 index is increased and linoleic acid is decreased in obesity-prone rats fed a high-fat diet. Lipids Health Dis.

[26] Cohen P, Ntambi JM, Friedman JM (2003). Stearoyl-CoA desaturase-1 and the metabolic syndrome. Curr. Drug Targets Immune Endocr. Metab. Disorders.

[27] Ntambi JM, Miyazaki M (2004). Regulation of stearoyl-CoA desaturases and role in metabolism. Prog. Lipid Res..

[28] Cheng X (2016). Critical role of miR-125b in lipogenesis by targeting stearoyl-CoA desaturase-1 (SCD-1). J. Anim. Sci..

[29] Likitmaskul S (2003). Increasing prevalence of type 2 diabetes Mellitus in Thai children and adolescents associated with increasing prevalence of obesity. J. Pediatr. Endocrinol. Metab..

[30] Van Gaal L, Mertens I, De Block C (2006). Mechanisms linking obesity with cardiovascular disease. Nature.

[31] Rocha VZ, Libby P (2009). Obesity, inflammation, and atherosclerosis. Nat. Rev. Cardiol..

[32] Krutzfeldt J, Stoffel M (2006). MicroRNAs: a new class of regulatory genes affecting metabolism. Cell Metab..

[33] Srinivasan S, Selvan ST, Archunan G, Gulyas B, Padmanabhan P (2013). MicroRNAs -the next generation therapeutic targets in human diseases. Theranostics.

[34] Esau C (2004). MicroRNA-143 regulates adipocyte differentiation. J. Biol. Chem..

[35] Meerson A (2013). Human adipose microRNA-221 is upregulated in obesity and affects fat metabolism downstream of leptin and TNF-α. Diabetologia.

[36] Xu N (2012). MicroRNA-125b down-regulates matrix metallopeptidase 13 and inhibits cutaneous squamous cell carcinoma cell proliferation, migration, and invasion. J. Biol. Chem..

[37] Iorio MV (2005). MicroRNA gene expression deregulation in human breast cancer. Can. Res..

[38] Vriens MR (2012). MicroRNA expression profiling is a potential diagnostic tool for thyroid cancer. Cancer.

[39] Henson BJ, Bhattacharjee S, Odee DM, Feingold E, Gollin SM (2009). Decreased expression of miR-125b and miR-100 in oral cancer cells contributes to malignancy. Genes Chromosom. Cancer.

[40] Nishida N (2011). MicroRNA miR-125b is a prognostic marker in human colorectal cancer. Int. J. Oncol..

[41] Han Y (2013). Hsa-miR-125b suppresses bladder cancer development by down-regulating oncogene SIRT7 and oncogenic long non-coding RNA MALAT1. FEBS Lett..

[42] Shi XB (2011). <em>miR-125b</em> promotes growth of prostate cancer xenograft tumor through targeting pro-apoptotic genes. Prostate.

[43] Liu L (2011). miR-125b suppresses the proliferation and migration of osteosarcoma cells through down-regulation of STAT3. Biochem. Biophys. Res. Commun..

[44] Ma Y, Tian Z, Wei Z (2012). CirculatingmiR-125bis a novel biomarker for screening non-small-cell lung cancer and predicts poor prognosis. J. Cancer Res. Clin. Oncol..

[45] Huang K, Dong S, Li W, Xie Z (2013). The expression and regulation of microRNA-125b in cancers. Acta Biochim. Biophys. Sin..

[46] Horii T, Hatada I (2014). Genome engineering using the CRISPR/Cas system. World J. Med. Genet..

[47] Donohoue PD, Barrangou R, May AP (2017). Advances in industrial biotechnology using CRISPR-cas systems. Trends Biotechnol..

[48] Shinmyo Y (2016). CRISPR/Cas9-mediated gene knockout in the mouse brain using in utero electroporation. Sci. Rep..

[49] Wettstein R, Bodak M, Ciaudo C (2015). Generation of a knockout mouse embryonic stem cell line using a paired CRISPR/Cas9 genome engineering tool. Methods Mol. Biol..

[50] Kurowska-Stolarska M (2017). MicroRNA-34a dependent regulation of AXL controls the activation of dendritic cells in inflammatory arthritis. Nat. Commun..

[51] Wang H, La Russa M, Qi LS (2016). CRISPR/Cas9 in genome editing and beyond. Annu. Rev. Biochem..

[52] Mendell JT, Olson EN (2012). MicroRNAs in stress signaling and human disease. Cell.

[53] Patrick DM (2010). Stress-dependent cardiac remodeling occurs in the absence of microRNA-21 in mice. J. Clin. Invest..

[54] Carrer M (2012). Control of mitochondrial metabolism and systemic energy homeostasis by microRNAs 378 and 378. Proc. Natl. Acad. Sci. U.S.A..

[55] Liu P (2017). Blocking FSH induces thermogenic adipose tissue and reduces body fat. Nature.

[56] Than NN, Newsome PN (2015). A concise review of non-alcoholic fatty liver disease. Atherosclerosis.

[57] Daniels SR (2005). Overweight in children and adolescents pathophysiology, consequences, prevention, and treatment. Circulation.

[58] Hu X (2015). Down-regulation of the miR-543 alleviates insulin resistance through targeting the SIRT1. Biochem. Biophys. Res. Commun..

[59] Yu Y (2018). Adipocyte-derived exosomal MiR-27a induces insulin resistance in skeletal muscle through repression of PPARγ. Theranostics.

[60] Wu J (2020). Hepatic exosome-derived miR-130a-3p attenuates glucose intolerance via suppressing PHLPP2 gene in adipocyte. Metab. Clin. Exper..

[61] Sanna F, Margaritis M, Antoniades C (2017). Perivascular adipose tissue as an endocrine organ: the role of statins. Curr. Pharm. Des..

[62] Stryjecki C (2012). Enzymatic activity and genetic variation in SCD1 modulate the relationship between fatty acids and inflammation. Mol. Genet. Metab..

[63] Zhang M, Sun W, Zhou M, Tang Y (2017). MicroRNA-27a regulates hepatic lipid metabolism and alleviates NAFLD via repressing FAS and SCD1. Sci. Rep..

[64] Chen Y-S (2020). MiR-1908/EXO1 and MiR-203a/FOS, regulated by scd1, are associated with fracture risk and bone health in postmenopausal diabetic women. Aging.

[65] El Helou R (2017). miR-600 acts as a bimodal switch that regulates breast cancer stem cell fate through WNT Signaling. Cell Rep..

[66] Zhang S (2016). DNA methylation landscape of fat deposits and fatty acid composition in obese and lean pigs. Sci. Rep..

[67] Miyazaki M, Bruggink SM, Ntambi JM (2006). Identification of mouse palmitoyl-coenzyme A Δ9-desaturase. J. Lipid Res..

[68] Miyazaki M (2009). Stearoyl-CoA desaturase-1 deficiency attenuates obesity and insulin resistance in leptin-resistant obese mice. Biochem. Biophys. Res. Commun..

[69] Biddinger SB (2005). Effects of diet and genetic background on sterol regulatory element-binding protein-1c, Stearoyl-CoA Desaturase 1, and the development of the metabolic syndrome. Diabetes.

[70] Cedernaes J, Alsiö J, Västermark A, Risérus U, Schiöth HB (2013). Adipose tissue stearoyl-CoA desaturase 1 index is increased and linoleic acid is decreased in obesity-prone rats fed a high-fat diet. Lipids Health Dis..

[71] Cohen P, Ntambi JM, Friedman JM (2003). Stearoyl-CoA desaturase-1 and the metabolic syndrome. Curr. Drug Targets Immune Endocr. Metabol. Disord..

[72] Tang YF (2009). Expression of miR-31, miR-125b-5p, and miR-326 in the adipogenic differentiation process of adipose-derived stem cells. OMICS J. Integr. Biol..

[73] Li HY (2012). Identification and comparison of microRNAs from skeletal muscle and adipose tissues from two porcine breeds. Anim. Genet..

[74] Liu X (2017). miR-192-5p regulates lipid synthesis in non-alcoholic fatty liver disease through SCD-1. World J. Gastroenterol..

[75] Xiao T (2015). Long noncoding RNA ADINR regulates adipogenesis by transcriptionally activating C/EBPα. Stem Cell Rep..

[76] Chen C (2005). Real-time quantification of microRNAs by stem–loop RT–PCR. Nucleic Acids Res..

