# Supplementary material for: MiR-125b-2 knockout increases high-fat diet-induced fat accumulation and insulin resistance
Source: Sci Rep. 2020 Dec 15;10:21969. doi: 10.1038/s41598-020-77714-7 (PMC7738482; doi:10.1038/s41598-020-77714-7)
Supplement: Supplementary file 1 — Supplementary Information. [file 41598_2020_77714_MOESM1_ESM.docx]

**Supplemental**

**Supplemental Table1: Composition and Nutritional Levels of Normal and High Fat Diets**

| Ingredients  (g/kg) | Normal diet | HFD | Nutritional level | Normal diet  mass ratio % | Normal diet  Energy Ratio % | HFD  mass ratio % | HFD  Energy Ratio % |
| --- | --- | --- | --- | --- | --- | --- | --- |
| casein | 200 | 200 | crude protein | 19.2 | 20 | 26.2 | 20 |
| L-Cystine | 3 | 3 | carbohydrate | 67.3 | 70 | 26.3 | 20 |
| corn starch | 315 | 0 | Fat | 4.3 | 10 | 34.9 | 60 |
| Maltodextrin | 35 | 125 | Total | 90.8 | 100 | 87.4 | 100 |
| sucrose | 350 | 68.8 |  |  |  |  |  |
| cellulose | 50 | 50 |  |  |  |  |  |
| soybean oil | 25 | 25 |  |  |  |  |  |
| lard | 20 | 245 |  |  |  |  |  |
| Mineral AIN-93 | 35 | 35 |  |  |  |  |  |
| Vitamin AIN-93 | 10 | 10 |  |  |  |  |  |
| Choline chloride | 2.5 | 2.5 |  |  |  |  |  |
| Total | 1045.5 | 764.3 |  |  |  |  |  |

**Supplemental Table2: Primer sequences for RT-PCR**

| mRNA | Sequences(5’ to 3’) |
| --- | --- |
| β-actin | F: TAAAGACCTCTATGCCAACACAGT |
|  | R: CACGATGGAGGGGCCGGACTCAT |
| SCD-1 | F: TGGGTTGGCTGCTTGTG |
|  | R: GCGTGGGCAGGATGAAG |
| PPARγ | F: TCGCTGATGCACTGCCTATG |
|  | R: GAGAGGTCCACAGAGCTGATT |
| C/EBPα | F: ATAGACATCAGCGCCTACAT |
|  | R: TCCCGGGTAGTCAAAGTCAC |
| U6 | F：CTCGCTTCGGCAGCACA |
|  | R：AACGCTTCACGAATTTGCGT |

**Figures and figure legends**


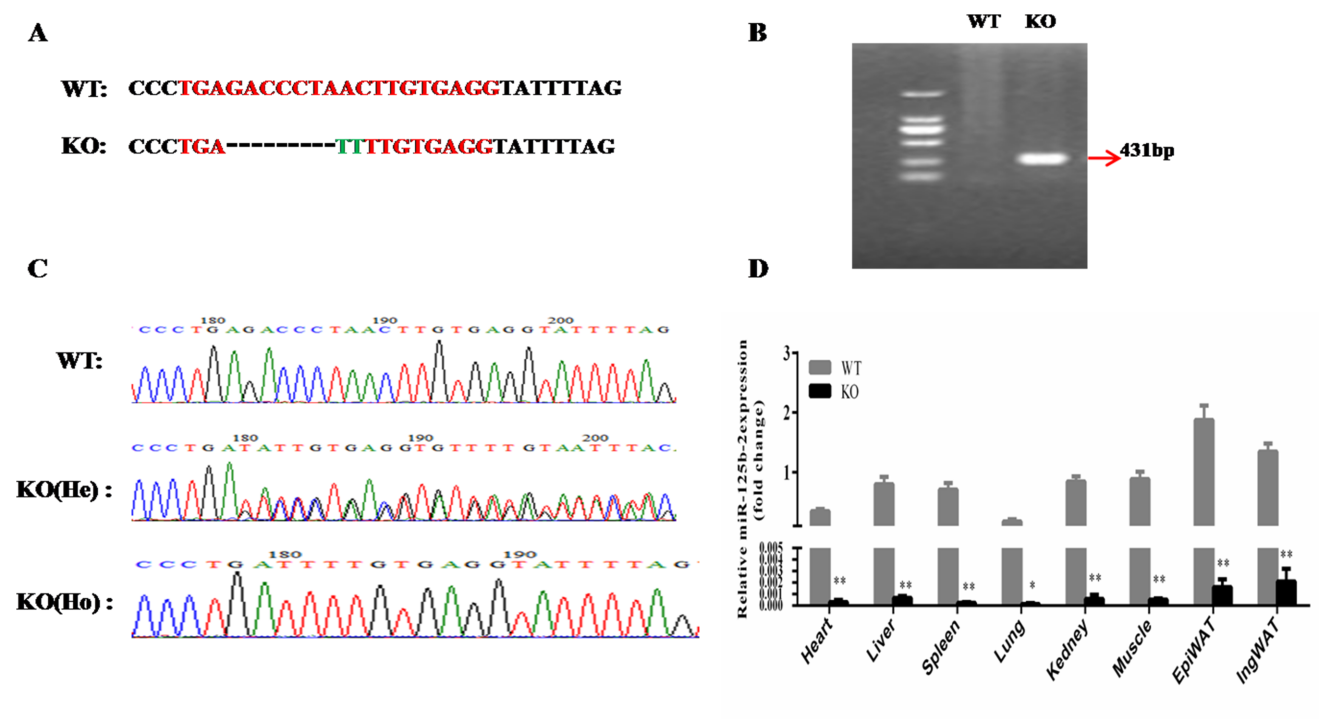


**Supplemental Figure1.** Identification miR-125b-2 knockout mice. (A) Gene sequence alignment of WT and miR-125b-2KO mice. (B) The electrophoresis results of genotyping of WT and miR-125b-2 KO homozygote mice. (C) Peak shape comparison of WT mice, miR-125b-2 heterozygote, and miR-125b-2KO mice. (D) Analysis of the expression levels of miR-125b-2 in WT mice and KO mice (miR-125b-2KO). **P* < 0.05, ***P* < 0.01.


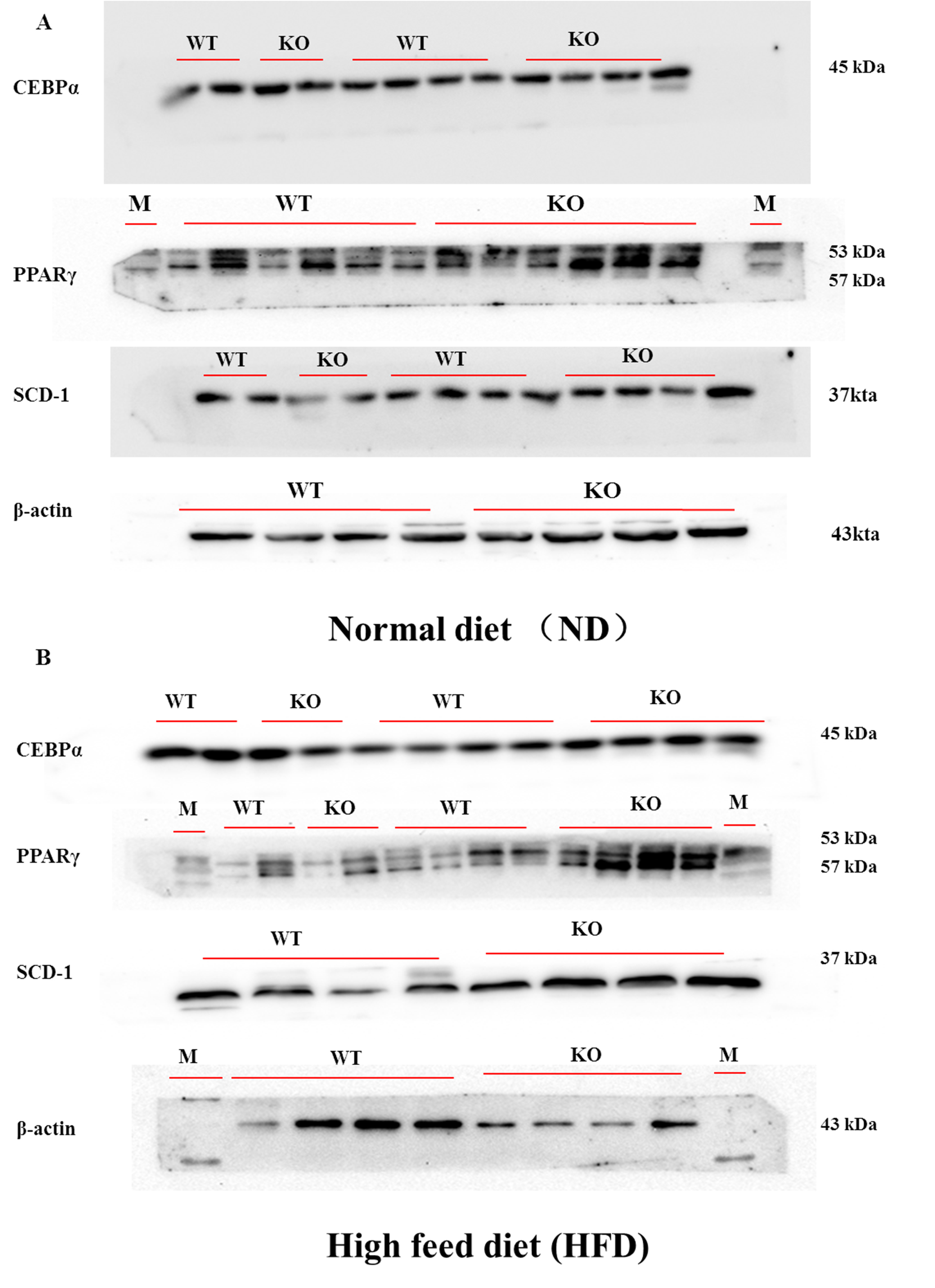


**Supplemental Figure 2.** Expression levels of C/EBPα, PPARγ, and SCD-1 protein in WT and KO mice fed a normal diet (ND) and a high fat diet (HFD). (A) Expression levels of C/EBPα, PPARγ and SCD-1 protein in WT and KO mice fed a ND. (B) Expression levels of C/EBPα, PPARγ and SCD-1 protein in WT and KO mice fed a HFD. M：Molecular weight markers indicating the molecular weights represented on each western blot membrane. n = 8 mice per group.
